# The Effects of Overhang Forming Direction on Thermal Behaviors during Additive Manufacturing Ti-6Al-4V Alloy

**DOI:** 10.3390/ma14133749

**Published:** 2021-07-05

**Authors:** Fan Wu, Zhonggang Sun, Wei Chen, Zulei Liang

**Affiliations:** 1 Power Beam Processing Laboratory, AVIC Manufacturing Technology Institute, Beijing 100024, China; wufandut@163.com; 2 Tech Institute for Advanced Material, Nanjing Tech University, Nanjing 210009, China; sunzgg@163.com (Z.S.); zuleiliang@163.com (Z.L.); 3 Shangi Institute for Advanced Material (Nanjing) Co., Ltd., Nanjing 210038, China

**Keywords:** selective laser melting, numerical simulation, overhang, forming direction, temperature evolution, distortion

## Abstract

Selective laser melting was recently introduced to fabricate complex parts that are likely to contain overhangs. Process parameters, scanning strategies, support structures, and fast prediction techniques are being frequently studied, but little information about overhang forming direction has been reported. In this study, the effects of overhang forming direction in the working plane on temperature evolution and distortion processes during selective laser melting of Ti-6Al-4V alloy were examined by means of numerical simulation and experimental verification. We found that forming from different directions can lead to significant differences in the early stage of the overhang building process, which were verified by both the simulations and the experiment. Some analyses were performed when enough layers had been built and suggestions are also given.

## 1. Introduction

Selective laser melting (SLM) is a promising technique that can offer the possibility of fabricating complex geometries with high precision by melting metallic powders using a high-energy laser beam in a “point-by-point scanning, track-by-track overlapping, layer-by-layer depositing” manner under an inert atmosphere [1]. The thermal gradient induced by the back-and-forth laser beam irradiation results in the thermal expansion and shrinkage of consolidated material, leading to thermal stress inside the parts and substrate, which can induce obvious thermal distortion or even failure [2]. It is worth noting that stress cracking usually happens on a particular side of a selective laser-melted complex part and there is a preferred forming direction in the working plane.

Detailed investigations on temperature evolution and transient thermal stress via experiments are underprepared in terms of the testing equipment due to the localized heating and rapid melting/solidification [3,4,5,6]. Numerical simulation is the most common method for revealing the thermal mechanisms of the SLM process [7,8,9,10,11,12]. Yang et al. [13] used design-expert software to optimize SLM parameters for maximum density and found that the most significant influence of the processing parameters on the density is powder thickness. Conti et al. [14] evaluated the effect of different process parameters on heat distribution and residual stresses via numerical simulation and put forward some valuable suggestions to improve the numerical model. Ashcroft et al. [15] used a thermo-mechanical model to better understand the effects of two different scan strategies on the generation of residual stress in SLM and their results showed a larger thermal gradient parallel to the scan vector. Cheng et al. [16] and Mumtaz et al. [17] examined the effects of scanning strategy in the SLM process via sequentially coupled numerical models and the results showed that the 90° or the 45° alternating scanning strategy has a smaller build direction deformation than in other cases. The deformation of overhanging is usually controlled by adding supports. Takaichi et al. [18] found that the fatigue strength of supported specimens was more than twice that of unsupported specimens. Zhang et al. [19] developed a feasible method of slimming support structures and used this method to eliminate the quantities of their support structures by 35% on average. Song et al. [20] proposed functionally graded support structures and found that these structures can maintain a higher temperature level than conventional uniform support structures, which is equivalent to an extra preheating effect and can also reduce distortion. It is extremely challenging to predict the distortion of a practical SLMed part if each single track is taken into account using conventional modeling methods. Guo et al. [21] and David et al. [22], respectively, developed a fast model to effectively predict the residual stress and part distortion with a reasonable accuracy. In addition, Krawiec [23] and Krawczyk [24] evaluated the surface topography of additive manufactured parts and studied the effect of face turning. Most previous works focused their attention on process parameters, scanning strategies, support structures, fast prediction techniques, or surface topography. Little information about the effects of overhang forming direction on thermal behaviors has been reported.

It is well known that the geometry of a SLMed part has an important influence on the final quality. An in-depth understanding of distortion process of overhangs and their associated thermal mechanisms can help to design support structures and improve the success rates of fabricating complex parts. In this study, two sequentially coupled thermo-mechanical simulations on T-shape overhang structure were run using COMSOL Multiphysics software (5.5, 2019, COMSOL, Stockholm, Sweden) to reveal the thermal behaviors (including heat distribution on selected overhang structure and the process of deformation) when forming a Ti-64 part from different directions. High thermal stress usually occurs in SLMed Ti-64 alloy due to the low heat conductivity (−20 W/m·K) and resulting in a high thermal gradient [25,26]. A moving volumetric heat source and some important boundary conditions are imposed in the models. Heat conduction, heat convection, latent heat of fusion/solidification, and thermal expansion/shrinkage are also taken into account. Unsupported T-shape Ti-64 parts were fabricated to validate the simulations.

## 2. Simulation and Experiment

### 2.1. Thermo-Mechanical Simulation

**Figure 1:**
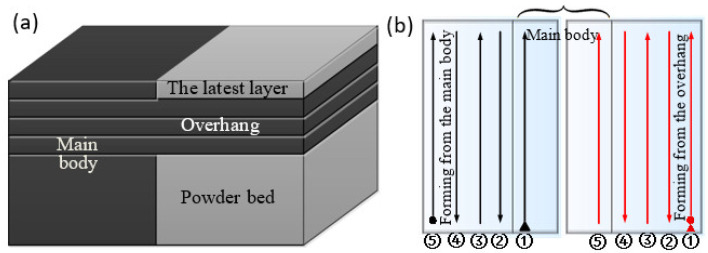
(**a**) Diagram of coupled thermo-mechanical model; (**b**) Laser scanning strategy and overhang forming direction.

The T-shape overhang, laser scanning strategy, and forming directions are shown in Figure 1. The overhang was built using three layers. The length and width of the overhang were 0.48 and 1.1 mm, respectively, as shown in Figure 1a. The laser scans the latest layer from the overhang side in a simulation and scans from the main body side in another simulation, are shown in Figure 1b. The starting points of laser processing are marked with inverted triangles in both simulations. Every track is numbered according to the scanning sequence, as shown in Figure 1b.

The ambient temperature was set to 303.15 K, the melting temperature of Ti-64 alloy was set to 1923 K, the solidification temperature was set to 1873 K, the latent heat of melting was set to 286 kJ/kg, the density of the bulk Ti-64 alloy was 4420 kg/m^3^, the thermal conductivity was set to 30 W/(m·K), and the specific heat capacity (c) varied with temperature, as shown in Equation (1).

(1) $$c=\{\begin{array}{c} \begin{array}{cc} \;\;\;\;\;\;483.04+0.215T\;\;\;\;\;\;\;\;\; & \;\;298<T<1268 \end{array}\\ \begin{array}{cc} \;\;\;\;\;\;\;\;\;412.7+0.18T\;\;\;\;\;\;\;\;\; & \;1268<T<1873 \end{array}\\ \begin{array}{cc} 749.84+1.6\left(T-1873\right) & \;\;1873<T<1923 \end{array} \end{array}$$

The thermal properties of the powder material were the same as the bulk materials, except that the thermal conductivity was 1% that of the bulk materials [27]. In the model, it is considered that only bulk materials expand when heated, and liquid materials almost do not expand. The coefficient of thermal expansion, γ, of Ti-64 is shown in Equation (2); Poisson’s ratio was set to 0.34, and the Young’s modulus and yield strength change with temperature are as shown in Table 1.

(2) $$\gamma =\{\begin{array}{c} \begin{array}{cc} \;\;\;\;\;\;\;\;\;\;\;\;\;\;\;\;\;\;\;\;\;\;1.68\;\;\;\;\;\;\;\;\;\;\;\;\;\;\;\;\;\;\;\;\;\;\; & 298<T<1898 \end{array}\\ \begin{array}{cc} 1.68-0.00026\left(T-1898\right) & \;\;\;\;\;\;1898<T<5000 \end{array} \end{array}$$

In order to reduce the amount of calculations, the top region of the powder layer was divided into small grids with the same size using “mapping + scanning”, and the cell size was 55 μm × 30 μm × 15 μm. Other regions were divided into tetrahedral meshes of different sizes in the form of a “free tetrahedron + fine mesh”. The process parameters for both simulations are shown in Table 2.

The laser energy followed a Gaussian distribution on the top surface of the model and decreased exponentially in the building direction. The laser energy can be ignored at a certain depth, which was empirically set to 0.05 mm. A strong heat convection occurred between the melting pool and ambient gas; therefore, the heat convection is only set on the top surface. Heat transfer at model’s bottom and at the four sides can be assumed to be negligible for model simplification. The temperature of the bottom can be taken as constant since the bottom is in indirect contact with the substrate. Thermal expansion is thought to occur in the built main body, the overhang, and the latest layer. In addition, the bottom is set as the fixed constraint and other faces are free. The thermo-physical properties of Ti-64 powder can be calculated [27] based on those of bulk Ti-64 alloy, which can be found in [28]. There were five tracks to scan in both simulations, a track took 0.0009 s and the total heating time was 0.0045 s. The total cooling time was set to 0.1 s, which was enough to cool the parts to ambient temperature.

### 2.2. Validation Experiment

Unsupported T-shape Ti-64 parts were produced on a Renishaw AM250 machine (Gloucestershire, UK) using the same process parameters as the simulation. In the working plane, the laser zigzagged back and forth, forming a deposited layer from the right side of T-shape parts to the left. The length and width of the overhangs were 2 and 5 mm, respectively. The thicknesses were set to 0.15, 0.3, 0.5, 0.7, and 0.9 mm, respectively. The parts were separated from the substrate by wire-cutting and the maximum values of the final distortions were measured at a magnification of 50 times using a stereo microscope (Carl Zeiss AG, Jena, Germany).

## 3. Results

### 3.1. Temperature Evolution

**Figure 2:**
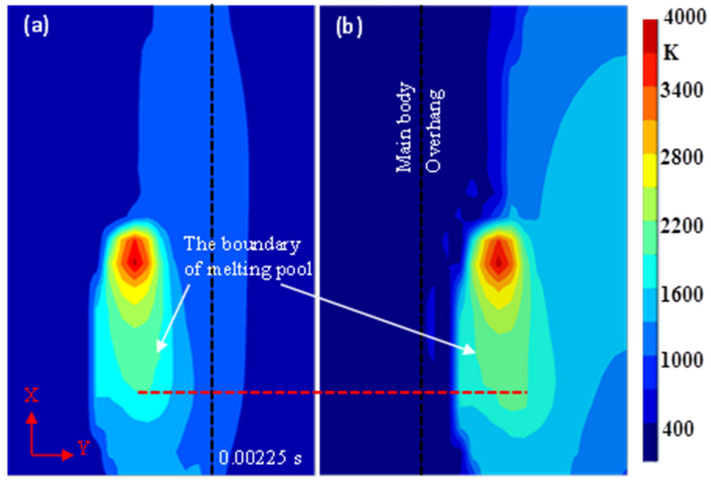
The temperature distribution on the X-Y plane (Z = 0, the top surface of the overhang) at 0.00225 s. (**a**) Forming from the main body; (**b**) Forming from the overhang.

The temperature distribution of the top surface at 0.00225 s is plotted in Figure 2.

**Figure 3:**
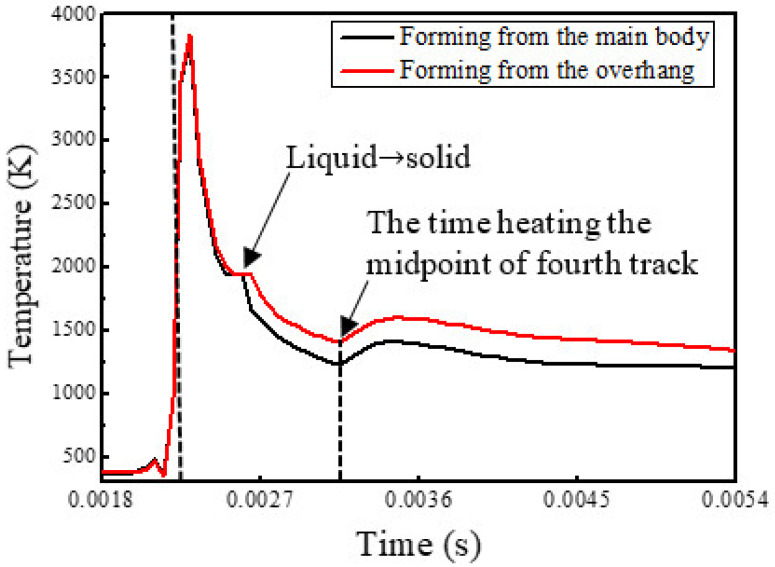
Temperature variations with time at the midpoints of both track 3.

When the time was 0.00225 s, the laser was irradiating the midpoints of both track 3. The two tracks have the same distance from the body in both simulations. The main body and the overhang are distinguished by the black dash lines in Figure 2. The temperature range at a point can be confirmed by the isothermal curves and the thermal gradient can be estimated by the isothermal density. The melting temperature of Ti-64 alloy was set to 1873–1923 K. The white arrows in Figure 2 indicate the 1900 K isotherm curves, within which are the melting pools. The melting pools are elongated in shape along the pathway of the moving heat source, and the length in Figure 2a is less than in Figure 2b, which are discerned by the red dashed line. Figure 2 also shows that the scanned area has a higher temperature than the unscanned area and that the temperature close to the heat source is higher than that of the surrounding area. Moreover, a lower temperature and a higher thermal gradient can be found in Figure 2a compared to Figure 2b, which is further illustrated in Figure 3.

Figure 3 shows the temperature histories of the midpoints of both track 3.

**Figure 4:**
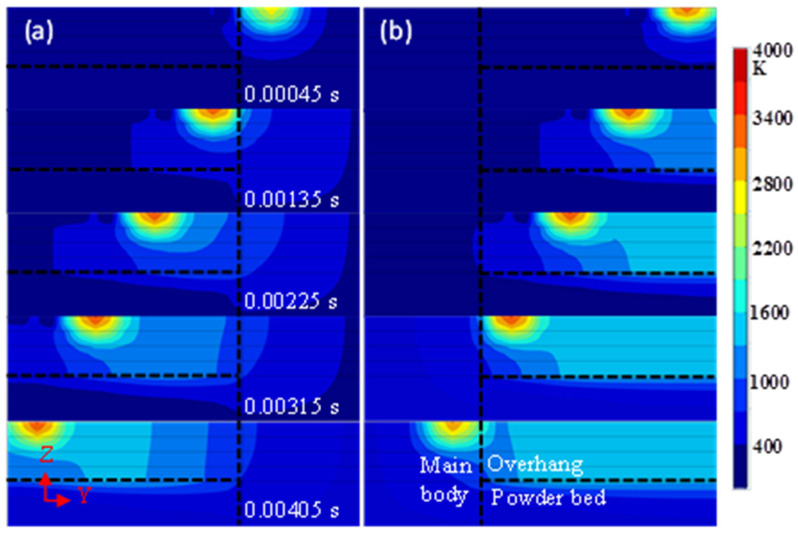
The transient temperature distribution on the Y-Z plane (X = 0.495 mm, the middle of the overhang in the X-direction) at 0.00045, 0.00135, 0.00225, 0.00315, and 0.00405 s, respectively. (**a**) Forming from the main body; (**b**) Forming from the overhang.

**Figure 5:**
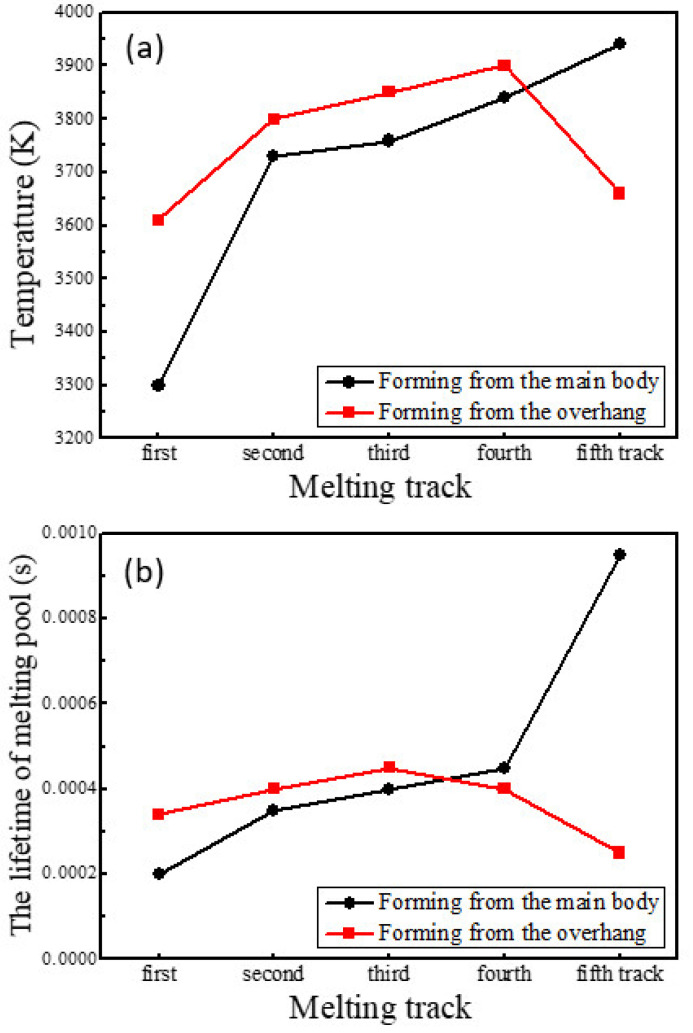
(**a**) The maximum temperatures at the midpoints of the five tracks; (**b**) The lifetime of the melting pool at the midpoints of the five tracks.

Before the heat source reached these points, their temperatures were close to ambient temperature; when the heat source was radiating the points, the temperatures increased rapidly and the peak values appeared behind the heat source. When heat source moved away, the temperature dropped rapidly. The estimated heating rate in Figure 3 is much larger than cooling rate, indicating that the heating processes are much faster than the cooling processes, which was also described in [29]. Especially, the cooling rates decreased rapidly when the melting pools were cooled to 1923 K due to the effect of the latent heat of solidification. The solidification processes continued until the temperatures dropped below 1873 K and then the temperature dropped sharply again. The effect of latent heat fusion is not enough to be noticeable due to the high heating rates. It is worth noting that, when forming the overhang and the temperature was below 1873 K, the temperature was higher and the cooling rate was lower than the temperature when forming the main body. When the midpoints of both track 4s are being heated, the temperatures increased slightly and then dropped slowly. The maximum temperatures and the lifetime of the melting pool were different in both simulations and the differences are further revealed in Figure 4 and Figure 5.

**Figure 6:**
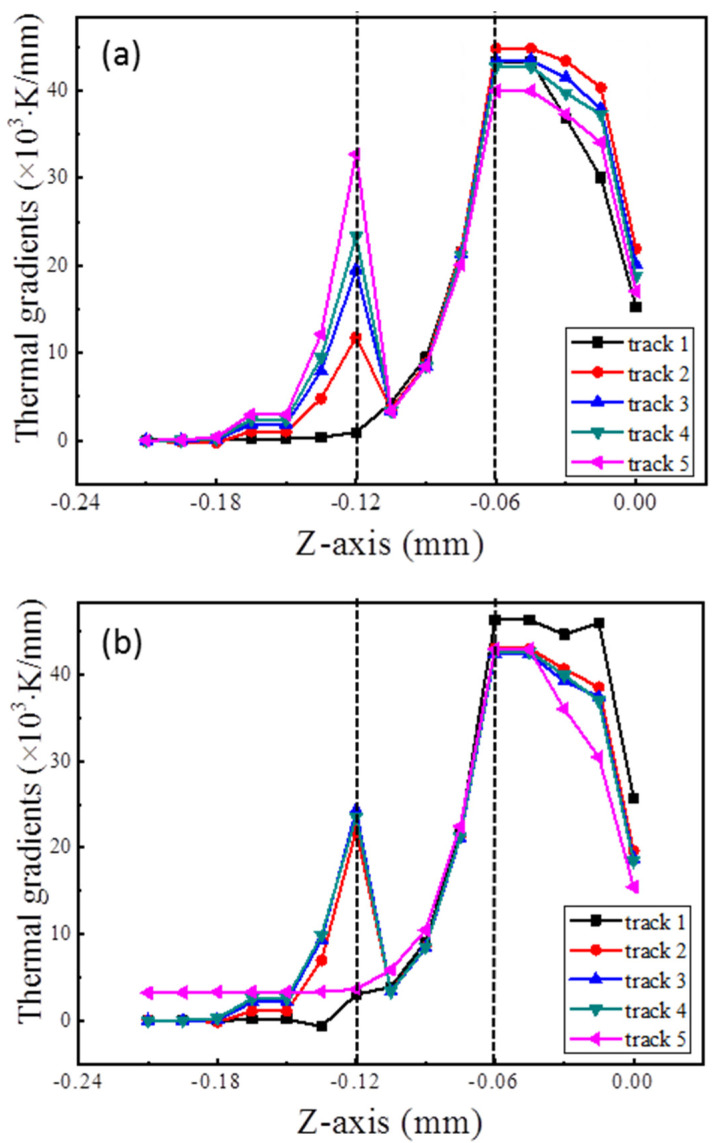
The distribution of thermal gradients in the *Z*-axis (X = 0.495 mm, Y = 0 mm, 0.12 mm, 0.24 mm, 0.36 mm and 0.48 mm, respectively, from track 1 to track 5 in both simulations). (**a**) Forming from the main body; (**b**) Forming from the overhang.

Figure 4 shows the transient temperature distribution on the transverse midsection of the model in both simulations at times 0.00045, 0.00135, 0.00225, 0.00315, and 0.00405 s, respectively. At these times, the heat source is heating the midpoints of the five tracks. The maximum temperatures and the lifetime of the melting pools at these points are presented in Figure 5 and the temperature gradients in the building direction (*Z*-axis) are shown in Figure 6.

During forming from the main body, there is a significant increase in the maximum temperature at the midpoint of track 2 compared with the midpoint of track 1 and then the value increased slowly; the lifetime of the melting pool also increased, especially at the midpoint of track 5. In another case, the maximum temperature increased gradually at the midpoints of the first four tracks and decreases significantly at the midpoint of track 5; the lifetime of the melting pool was extended at the midpoints of the first three tracks and shortened at the midpoints of the latter two tracks. It can be seen from Figure 5 that when forming from the main body, the values of the maximum temperature and the lifetime of the melting pool were less than when initially forming the overhang, and were greater later.

Thermal gradient is essential during SLM processes as it, not only has strong influence on the main direction of distortion, but also determines the distortion degree. In both simulations, the maximum thermal gradients of the built layers in the building direction (*Z*-axis) were greater than 40 × 10^3^ K/mm, less than 25 × 10^3^ K/mm in the longitudinal direction (*X*-axis), and less than 10 × 10^3^ K/mm in the transverse direction (*Y*-axis). Hence, the thermal gradients on the *Z*-axis were the most important. The distribution of the thermal gradient in the lines (which paralleled the *Z*-axis and passed through the midpoints of the five tracks) in both simulations are shown in Figure 6.

In both simulations, the temperature gradient peaked at a depth of 0.06 mm from the top surface. It is believed that the depth of the melting pool was about 0.06 mm, according to Figure 3. There is also a maximal value for the temperature gradient at the boundary between the powder bed and the built layers, except if there was no powder bed below the melting pool (such as the black curve in Figure 6a and the pink curve in Figure 6b). During forming of the main body, the building-directional thermal gradient in the built layers degraded gradually with the increasing number of tracks, except the first one, which was relatively low. In another case, the thermal gradient in track 1 was higher than the others which did not show much difference in terms of maximum value. Specifically, the pink curve in Figure 6a is below that of its counterpart in Figure 6b.

### 3.2. Distortion Process

**Figure 7:**
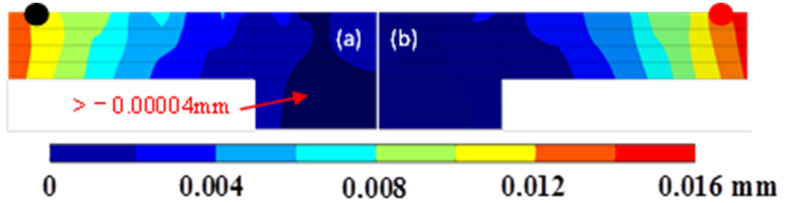
The final displacement distribution on the Y-Z plane (X = 0.495 mm). (**a**) Forming from the main body; (**b**) Forming from the overhang.

In both simulations, the displacement in the building direction was greater than 0.0122 mm, less than 0.0068 mm in the longitudinal direction, and less than 0.0061 mm in the transverse direction. The displacement distribution in the building direction is plotted in Figure 7.

**Figure 8:**
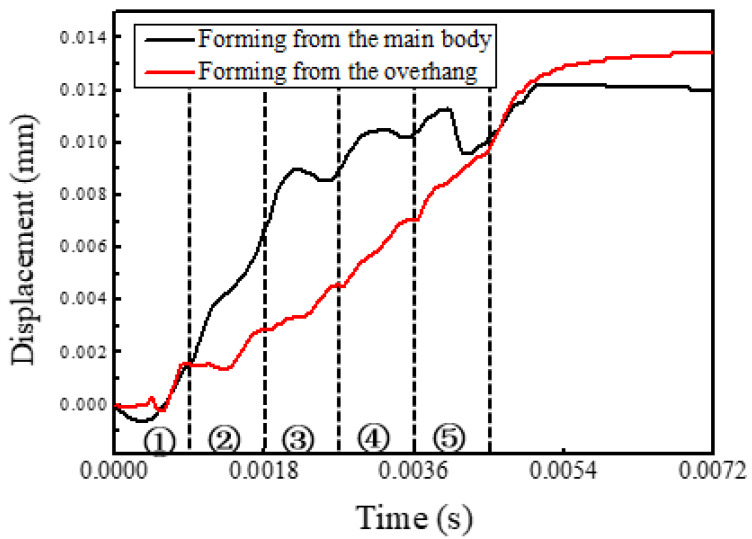
The displacement variations in the building direction with time at the midpoints of both track 5.

To characterize the process of the distortion, the measurement points indicated by the black dot and red dot in Figure 7 were created at the midpoints of both track 5s. The displacement variations of both dots in the building direction are presented in Figure 8. The closer the heat source was to the measurement points, the more obvious both of the upward and downward displacements will be; when the heat source is far from the measurement points, only the upward displacement is obvious. During forming of the main body, the displacement increased rapidly at first and then leveled off on the whole. In another case, the displacement increased linearly in general and overtook the former case eventually.

### 3.3. Experimental Investigation

**Figure 9:**
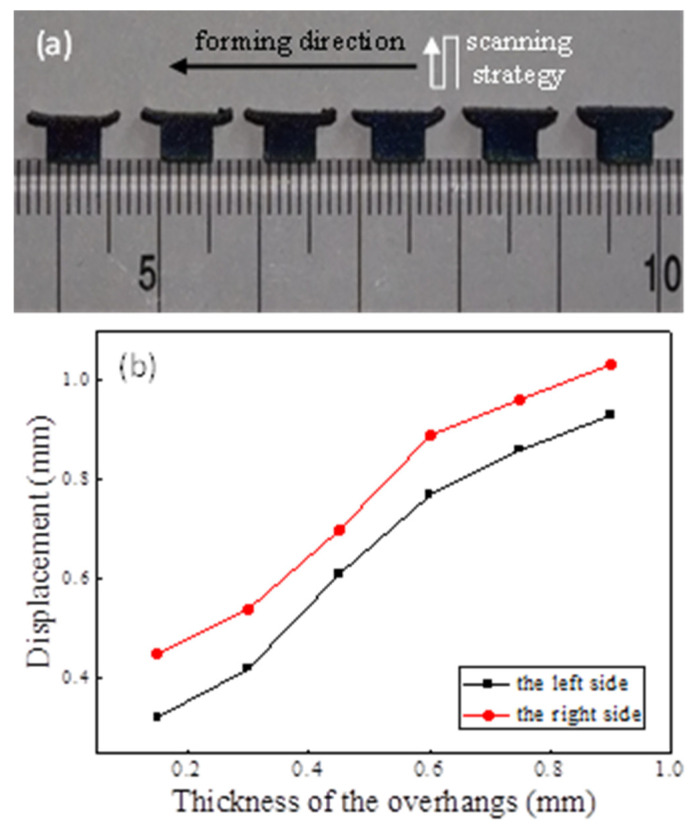
(**a**) The as-fabricated T-shape overhangs (length = 2 mm, width = 5 mm, thickness = 0.15, 0.3, 0.5, 0.7, and 0.9 mm, respectively); (**b**) Displacements of the edges of the overhangs in the building direction.

The deformed T-shape overhangs were photographed with a stereo microscope at 50 times, and then the true displacements were calculated using the ratio of the scale to the measured size. The as-fabricated T-shape overhangs, the laser scanning strategy, and the forming direction on the working plane are shown in Figure 9a and the maximum displacements of the overhangs in the building direction are shown in Figure 9b. It can be seen that the distortion of the right overhang is greater than that of the left one in a particular part, and the distortion increases with the thickness increasing.

## 4. Discussion

Temperature plays a significant role in the distortion process, which depends on the thermal expansion and shrinkage of local material. Understanding the effect of forming direction on temperature evolution can help us analyze the distortion process.

During the SLM process, a small high-energy laser moves rapidly and local powder material is heated and melts to form a melting pool and the melting pool cools into bulk material. In fact, the laser can be treated as a volumetric heat source which obeys the Gaussian distribution on the working plane and decreases as it penetrates into the powder bed [30,31]. A high thermal gradient develops within the melting pool since the laser energy is not evenly distributed, as shown in Figure 2 and Figure 6. The temperature of the surrounding material is much lower than that of the melting pool. Therefore, the peak value occurs at the boundary of the melting pool and the thermal gradient degrades gradually in the solidified region because of the high thermal conductivity, as shown in Figure 6.

Due to the small contact area between particles, the thermal conductivity of powder can be supposed to be 1% of the solid thermal conductivity [27,32]. In addition, the unsupported overhang is not fixed to the substrate in the model, leading to a lower cooling rate than the main body. Thus, the overhang will maintain a higher temperature than the main body as the process parameters are the same, as shown in Figure 2 and Figure 3, which is equivalent to preheating with a higher temperature for the scanning of the next track. When the heat source moves away from the main body, the cooling rate decreases and such a preheating effect enhances, and vice versa, as shown in Figure 2, Figure 3 and Figure 4. Additionally, at the same cooling rate, the preheating effect of the latest scanned track can increase the temperature of melting pool in the scanning track, resulting in further enhancements of the preheating effect on the next track. As a result, when forming on the main body, the temperature and the lifetime of the melting pool at the midpoints of the tracks increase with the increasing number of tracks; when forming from another side, the temperature and the lifetime increase initially and the decrease, as shown in Figure 4 and Figure 5.

**Figure 10:**
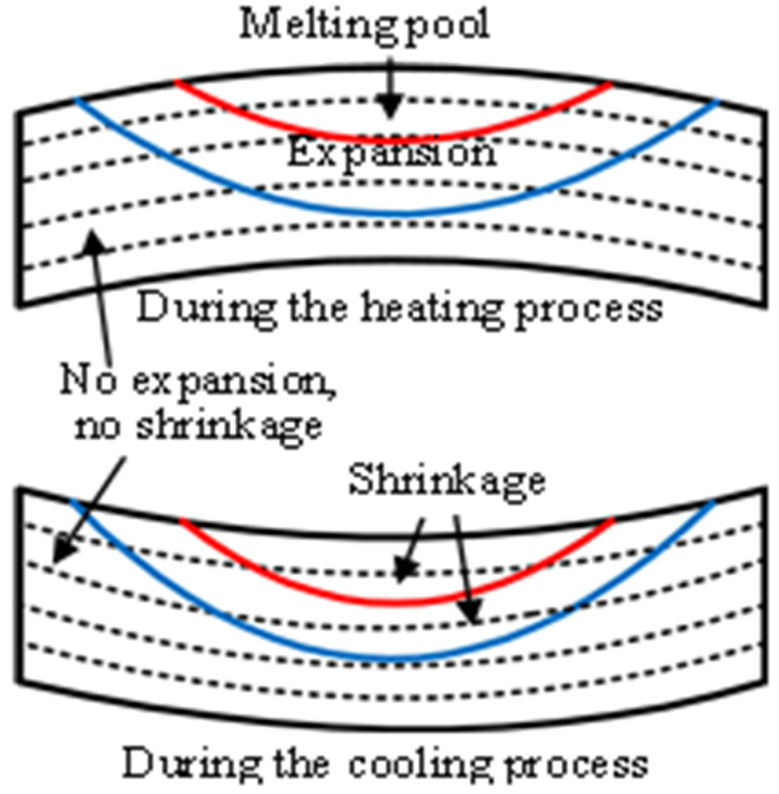
Schematic diagram of distortion during the SLM process.

Figure 10 [33,34] shows a schematic diagram of the distortion during the SLM process.

Due to the rapid heating of the heat source on the latest layer, a melting pool develops and the material below the melting pool is heated and expands. The region near the melting pool, which has a higher temperature, has more expansion. The expansion exerts a downward component force and an outward component force on the surrounding material. When the material yield stress, which is lowered due to the elevated temperature, is reached, a counter bending away from the melting pool will be perceived. During the cooling process, the heat source has left and the temperature drops rapidly. The melting pool solidifies and then shrinks and the material below the melting pool also shrinks. The more the temperature drops, the more obvious the shrinkage trend becomes. The shrinkage exerts an upward component force and an inward component force. Finally, the upper layers become shorter than the bottom layers due to the presence of the melting pool, which was verified by the simulations and the experiment, as shown in Figure 7, Figure 8 and Figure 9.

A high thermal gradient means more expansion/shrinkage in regions of elevated/reduced temperatures, resulting in a high thermal stress/distortion. The thermal gradients in the building direction are higher than those in the other two, indicating the largest thermal stresses/distortion in this direction. Moreover, the dimension of the overhang in the *Z*-axis is the lowest in the model. Therefore, the maximum displacement occurs in the building direction and the displacement increment increases with increasing thickness, as shown in Figure 9.

Supported overhangs can more effectively dissipate heat during building processes, which leads to a higher thermal gradient than those of unsupported overhangs. Support structures can degrade thermal distortion and prevent micro-cracks caused by thermal distortion, ensuring dimensional precision and mechanical properties [18]. When enough layers have been formed, there is little difference in the cooling rate and the last scanned area in a layer has the highest temperature due to the preheating effect. A high temperature will result in a high thermal gradient and a large thermal distortion/stresses [35]. These analyses suggest that forming from the main body can reduce thermal stress/distortion at the beginning of the building process and the forming direction should change frequently to avoid thermal stress concentration in a specific direction when enough layers are formed.

## 5. Conclusions

In this study, the thermal behaviors of forming a layer of a T-shape overhang from different directions were simulated and some unsupported T-shape parts were built using the Renishaw AM250 machine.

1. Forming from the main body can decrease the cooling rate of melting pool gradually, which leads to a lowered thermal gradient compared with forming from the overhang.
2. The overhang formed from the main body has a low thermal distortion, which was verified by both simulations and an experiment.
3. The further analyses suggest that the forming direction should be changed frequently to avoid thermal stress concentration in a specific side when enough layers have been formed.

## Figures and Tables

**Table 1:** Young’s modulus and yield strength of Ti-64 alloy.

Temperature (K)	Young’s Modulus (GPa)	Yield Strength (MPa)
297	125	1000
367	110	730
478	100	630
590	90	525
701	80	-
812	74	-
923	55	300
1034	27	-
1145	20	45
1367	5	5
1873	0.1	0.1

**Table 2:** The process parameters of both simulations and the experiment.

Parameter	Value
Laser power	200 W
Radius of laser spot	0.07 mm
Scanning point distance	0.055 mm
Laser residence time	5 × 10^−5^ s
Hatch spacing	0.12 mm
Layer thickness	0.03 mm

## Data Availability

All the data is available within the manuscript.
